# Time-restricted eating for patients with diabetes and prediabetes: A systematic review

**DOI:** 10.3389/fnut.2022.1025919

**Published:** 2022-11-03

**Authors:** Xiaoxiao Lin, Yihong Guan, Guomin Wu, Jinyu Huang, Shuai Wang

**Affiliations:** ^1^The Fourth School of Clinical Medicine, Zhejiang Chinese Medical University, Hangzhou, China; ^2^Department of Cardiology, Affiliated Hangzhou First People’s Hospital, Zhejiang University School of Medicine, Hangzhou, China; ^3^Department of Translation Medicine Center, Affiliated Hangzhou First People’s Hospital, Zhejiang University School of Medicine, Hangzhou, China

**Keywords:** diabetes, prediabetes, time-restricted eating (TRE), insulin sensitivity, a systematic review

## Abstract

**Background:**

Several studies have explored the effect of time-restricted eating (TRE) on patients with diabetes and prediabetes. However, these studies have not been analyzed and summarized as a whole. We conducted a systematic review to summarize and analyze all studies about the efficacy and safety of TRE for patients with diabetes and prediabetes.

**Methods:**

We conducted a comprehensive search of the Embase, PubMed and Cochrane databases and the time span was from inception to 1 May 2022. The Cochrane Collaboration’s Risk of Bias 2 (RoB2) and ROBINS-I tools were used to evaluate the quality of included studies. The effect of TRE on weight loss, insulin sensitivity, plasma glucose, and the safety of TRE were summarized and analyzed.

**Results:**

In total, 7 studies with 326 participants including 5 articles with 217 patients with diabetes and 2 articles with 109 patients with prediabetes were included. The TRE windows were from 4 to 10 h. The percentages of females ranged from 0 to 90%. The mean age ranged from 35.2 to 67.5 years, and most of patients adhered to TRE. All studies were assessed as high quality. TRE may result in weight loss, and improvements in the insulin sensitivity and plasma glucose, with no severe AEs.

**Conclusion:**

Time-restricted eating is a safe and feasible intervention, and may offer cardiovascular and metabolic benefits for patients with diabetes and prediabetes. Studies in this field, which should be viewed as important, are limited. Therefore, more high-quality studies are needed.

## Introduction

Time-restricted eating (TRE) is an emerging dietary intervention that is becoming increasingly popular (1). TRE has been demonstrated to ameliorate in obesity and metabolism. Previous studies have demonstrated that TRE was a promising dietary intervention to improving metabolic health and body composition in both overweight and non-overweight individuals (2–10).

Several studies have explored the effect of TRE on patients with diabetes and prediabetes, and demonstrated that TRE has the beneficial effects on 24-h plasma glucose, insulin sensitivity, weight loss, and glucose tolerance. For example, Che et al. (11) explored the effects of TRE over 12 weeks on weight changes and glycemic regulation in overweight patients with diabetes, and found that 10-h TRE could result in weight loss, improve blood glucose and insulin sensitivity, reduce the necessary dosage of hypoglycemic drugs, offer cardiovascular benefits and enhance the quality of life. Andriessen et al. (12) determined the clinical tolerability and biochemical effects of short-term TRE for patients with type 2 diabetes, and the results showed that short-term daily TRE may be a tolerable and safe dietary intervention and may improve outcomes of postprandial variability, fasting glucose and body weight for patients with T2DM. However, these studies were not analyzed and summarized as a whole. We conducted this systematic review to summarize and analyze all studies about the efficacy and safety of TRE for patients with diabetes and prediabetes.

## Methods

### Evidence before this study

We searched Pubmed, Embase, and Cochrane databases from inception to 1 May 2022, for previously published systematic reviews and meta-analyses about TRE for patients with prediabetes and diabetes, using the search terms “time restricted,” TRE, TRF, diabetes, prediabetes, meta-analysis, and systematic review, with no language restrictions. There was no systematic review and meta-analysis of TRE for patients with prediabetes and diabetes. Due to the importance of this topic, we performed this systematic review to summarize and analyze all trials of the effect of TRE for patients with diabetes or prediabetes.

### Search strategy

Our study was conducted according to and PRISMA criteria and Cochrane Collaboration guidelines (13, 14). We performed a comprehensive search for Embase, Pubmed, and Cochrane databases and the time span was from inception to 1 May 2022. The terms were used for searching: (“Type 2 Diabetes” OR “Diabetes type 2” OR diabetes OR “Type 2 Diabetic” OR T2DM OR T1DM OR “Type 1 Diabetes”) AND (“Intermittent Fasting” OR “time restricted feeding” OR “time restricted” OR “time-restricted eating” OR “time-restricted feeding” OR “time restricted eating” OR “time-restricted” OR TRE OR TRF). Clinical studies reported the efficacy and safety of TRE for patients with prediabetes and diabetes were included. The reference lists of relevant reviews and clinical trial registries were also manually searched. This process was conducted by two investigators independently (Wang and Lin), and discussed with the third reviewer (Huang).

### Inclusion and exclusion criteria

According to the PICOS principle, the inclusion criteria were made as follows: (P) Patients: patients with diabetes or prediabetes. (I) Interventions: TRE or eating window was under 10-h. (C) Control: without TRE. (O) Outcomes: the effect of TRE on weight loss, insulin sensitivity, plasma glucose, and the safety of TRE. (S) Study: clinical studies including RCTs and non-RCTs. The comments, editorials, letters to the editor, case reports, and conference abstracts were excluded.

### Quality appraisal

For RCTs, the risk of bias was evaluated according to the RoB2, which included six aspects: selective outcome, blinding, sequence generation, incomplete outcome data, the allocation concealment, and other biases (13). For non-RCTs, the ROBINS-I tool was used to identify seven domains of bias due to the selection of the reported result, deviations from intended interventions, confounding, missing and measurement of outcomes data, classification of interventions, and selection of participants into the study.

### Data extraction

Two reviewers (Wang and Lin) conducted data extraction independently, and recorded the information into three predesigned forms: (1) study and year, total number, design, TRE window, methods of tracking TRE and the type of patients. (2) Study and year, female (%), BMI (kg/m^2^), mean age, adherence to TRE, and antidiabetic drugs. (3) Study and year, main findings, and adverse effects (AEs), discussed with the third reviewer (Huang). All studies were summarized descriptively and assessed qualitatively.

## Results

### Literature search

**FIGURE 1:**
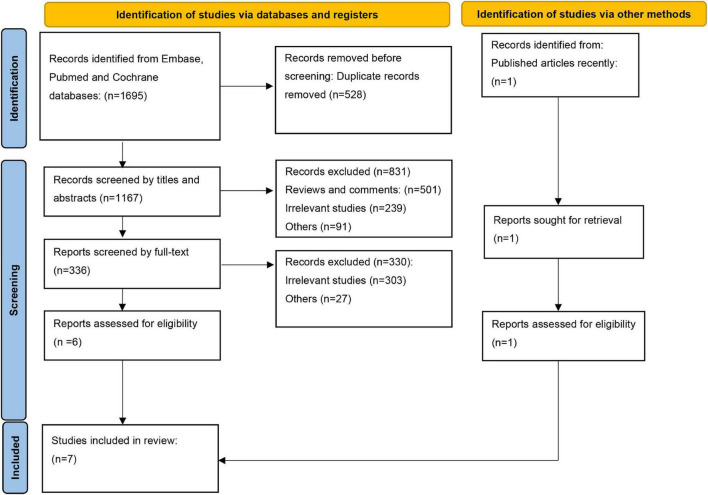
Search flow diagram.

In total, 1,695 articles were identified in the initial literature search. After excluding the duplications, 1,167 articles were screened by title and abstracts, and 336 full-text articles were reviewed. In addition, a recently published article (12) was included to make our study more comprehensive. In total, seven studies including five articles on patients with diabetes and two articles on patients with prediabetes, were included (11, 12, 15–19). The search flow is shown in Figure 1.

### Study characteristics and quality appraisal

**FIGURE 2:**
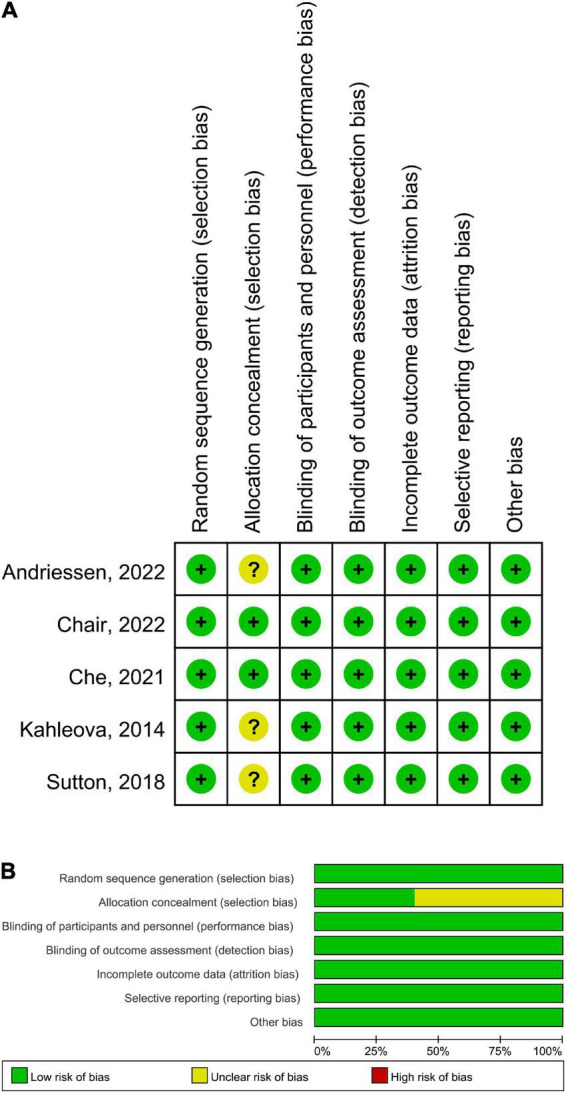
Risk of bias of RCTs by the Cochrane risk assessment tool. **(A)** Risk of bias graph. **(B)** Risk of bias summary.

A total of 326 participants containing 217 patients with diabetes and 109 with prediabetes were included, with a sample size ranging from 8 to 120. There were five RCTs and two non-randomized trials. The TRE windows were from 4 to 10 h. The percentages of female ranged from 0 to 90%. The mean age ranged from 35.2 to 67.5 years. Most of the patients were adherent to TRE. In patients with diabetes, the majority of antidiabetic drugs were metformin, DPP-4 inhibitors and SGLT2 inhibitors. The characteristics of the studies and patients are summarized in Tables 1, 2. For quality appraisal, the results are displayed in Figure 2 for RCTs and Table 3 for non-randomized trials, and all studies were assessed as high quality. In total, all studies were randomized for RCTs, and opaque envelopes were used for allocation concealment in two studies. For non-RCTs, all domains in two studies were assessed as low risk.

**TABLE 1:** The characteristics of studies.

Study, year	N	Design	TRE Window	Methods of tracking TRE	Type of patients
Arnason and Kerry (19)	10	Three-phase observational study (2-week baseline, 2-week intervention, 2-week follow up)	4–6 h eating window; 2 weeks	Self-reporting; remote food photography for a random 3-day food diary	T2DM (fasting glucose > 7.0 mmol, HbA1c > 6.5%, or OGTT > 11.0 mmol)
Andriessen et al. (12)	14	Randomized crossover trial	within a 10 h window no later than 18:00 h 10 weeks	A weekly phone call	T2DM, BMI > 25 kg/m
Chair et al. (15)	101	RCT (randomized into the ADF group, 18/8 TRF group, and control group)	8-h TRE; 3 weeks	Individualized menus and counseling; weekly telephone calls from nurse	Prediabetes
Che et al. (11)	120	RCT (TRE and control group)	10-h TRE (8 a.m.–6 p.m.); 12 weeks	A daily log was used to record	T2DM
Kahleova et al. (16)	54	RCT (randomized, open, crossover, single-center study)	breakfast (6–10 a.m.); lunch (12:00–4 p.m.) 12 weeks	All the meals were provided for one half of the participants; dietary record	T2DM
Parr et al. (17)	19	The non-randomized study	9 h TRE (10 a.m.–7 p.m.), 4 weeks	Written food diary or smartphone app to record all food entries. Photos of each food/beverage also taken using phone	T2DM (HbA1c > 6.5 and < 9%) and eating window > 12 h
Sutton et al. (18)	8	Randomized crossover trial	6 h TRE (no later than 14;00 h), 5 weeks	All meals provided and consumed under supervision	Prediabetes

TRE, Time-restricted eating; TRF, Time-restricted feeding; BMI, Body Mass Index; ADF, Alternate-day Fasting; T2DM, Diabetes Mellitus type 2; HbA1c, Glycosylated Hemoglobin, Type A1c; OGTT, Oral Glucose Tolerance Test; RCT, Randomized Clinical Trial.

**TABLE 2:** The characteristics of patients.

Study, year	Female (%)	BMI (kg/m^2^)	Mean age, years	Adherence to TRE	Antidiabetic drugs
Arnason and Kerry (19)	90%	36.9 ± 8.29	53.8 ± 9.11	80% adherent to TRE	Metformin; gliclazide; liraglutide.
Andriessen et al. (12)	50.00%	30.5 ± 3.7	67.5 ± 5.2	100% to TRE and control	Metformin only; metformin, gliclazide; others
Chair et al. (15)	63.30%	26.7 ± 2.0	35.2 ± 6.2	97% adherent to ADF; 100% adherent to TRE and control	None
Che et al. (11)	45.80%	26.3 ± 2.1	48.5 ± 9.4	90% adherent to TRE and 83.3% to control	OHA; insulin
Kahleova et al. (16)	46%	32.6 ± 4.9	59.4 ± 7.0	88.9% adherent to TRE and 85.2% to control	Metformin; DPP-4 inhibitors; sulfonylurea; glinides; thiazolidinedione; acarbose
Parr et al. (17)	52.60%	34.0 ± 5.0	50.0 ± 9.0	72% adherent to TRE	Metformin; SGLT-2 inhibitors; DPP-4 inhibitors
Sutton et al. (18)	0	32.2 ± 4.4	56.0 ± 9.0	100% to TRE and 98.9% to control	None

TRE, Time-restricted eating; ADF, Alternate-day Fasting; OHA, Oral hupoglycemic agents; DPP-4, Dipeptidyl peptidase-4; SGLT-2, Sodium-dependent glucose transporters 2.

**TABLE 3:** Quality appraisal for non-randomized trials.

Authors (year)	Confounding	Selection of participants	Classification of interventions	Deviations from intended interventions	Missing data	Measurement of outcomes	Selection of reported result
Arnason and Kerry (19)	Low	Low	Low	Low	Low	Low	Low
Parr et al. (17)	Low	Low	Low	Low	Low	Low	Low

### The efficacy and safety of time-restricted eating for patients with diabetes

The main findings and AEs are summarized in Table 4. In the included studies, Kahleova et al. (16) conducted an open, crossover, randomized and single-center study to investigate the effect of two meals (<10 h) compared to six meals on insulin resistance, hepatic fat content, beta cell function, and body weight for patients with diabetes. They found that eating only breakfast and lunch could increase oral glucose insulin sensitivity (OGIS), and reduce hepatic fat content (HFC), C-peptide, fasting plasma glucose, and body weight.

**TABLE 4:** The main findings and adverse effects (AEs).

Study, year	Main finding	AEs
Arnason and Kerry (19)	Short-term daily TRE may be a safe, tolerable, dietary intervention in T2DM patients that may improve key outcomes including body weight, fasting glucose and postprandial variability	No hypoglycemic events
Andriessen et al. (12)	TRE was a safe and feasible treatment, and effective to improve 24 h glucose homeostasis, with weight gain, but did not result in alterations in hepatic glycogen and insulin sensitivity.	No adverse events were observed
Chair et al. (15)	The 16/8 TRF achieved results in reducing weight and BMI and improving plasma glucose.	Three participants occasional feelings of hunger during fasting hours; No severe, adverse events were reported.
Che et al. (11)	10 h TRE could result in weight loss, reduced plasma glucose, and improved insulin sensitivity.	No any adverse events; No hypoglycemic events in the TRF group, one in the control group.
Kahleova et al. (16)	10 h TRE reduced body weight, plasma glucose and increased OGIS (oral glucose insulin sensitivity).	No adverse events were observed
Parr et al. (17)	4-weeks of 9-h TRE was feasible and achievable, but without effect on weight loss and 24 h glucose homeostasis.	No hypoglycemic or other adverse events
Sutton et al. (18)	6 h TRE improved insulin sensitivity and β-cell function, lower blood pressure, oxidative stress, evening appetite, and postprandial insulin, but have no effect on body weight and	No adverse events were observed

TRE, Time-restricted eating; TRF, Time-restricted feeding; BMI, Body Mass Index; T2DM, Diabetes Mellitus type 2; OGIS, Oral Glucose Insulin Sensitivity.

Che et al. (11) aimed to explore the effect of TRE in patients with diabetes, and their results suggested that 10-h restricted feeding could improve insulin sensitivity and blood glucose, reduce the weight and the dose of hypoglycemic drugs, enhance the quality of life, and offer cardiovascular benefits by lowing atherosclerotic lipid levels.

Parr et al. (17) conducted a post, non-randomized study to explore the feasibility of TRE for patients with diabetes. The primary outcomes were the indicators of feasibility including recruitment, retention, acceptability and safety, and the secondary outcomes were cognitive outcomes, glycemic control, psychological wellbeing and dietary intake. They found that TRE did not reduce body mass significantly and improve measures of glycemic control significantly. TRE did not improve or impair psychological wellbeing. They demonstrated that 4-weeks of TRE was feasible for patients with diabetes. Effects to support participants overcoming barriers to adherence and incorporating TRE in regular daily life could be beneficial.

Arnason et al. (19) performed a three-phase observational study to explore the biochemical, clinical and tolerability of TRE in patients with T2DM. They found that short-term daily TRE may be a tolerable and safe intervention for patients with diabetes and may improve key outcomes such as fasting glucose, postprandial variability and fasting glucose.

Andriessen et al. (12) investigated the effects of TRE on insulin sensitivity and hepatic glycogen levels in patients with diabetes. In their study, fourteen patients were included. The results demonstrated that 10-h TRE was an effective, safe and feasible method to improve glucose homeostasis but not insulin sensitivity or hepatic glycogen. There were no severe AEs in all studies.

### The efficacy and safety of time-restricted eating for patients with prediabetes

In Sutton’s study (18), the first trial to investigate whether TRE has benefits in humans without weight loss was conducted. They found early time-restricted feeding (eTRF) could increase insulin sensitivity, improve β-cell function, lower oxidative stress, blood pressure, and the desire to eat in the evening, and improve health even without weight loss. The eTRF was an effective strategy for treating both prediabetes and prehypertension which could improve some aspects of cardiometabolic health.

A recent study (15) aimed to examine the effects of TRE and alternate-day fasting (ADF) on blood glucose, weight loss, and lipid profiles in adults with prediabetes who were overweight/obese. Compared with those in the control group, the reductions in BMI, weight and waist circumference in the TRE and ADF groups were more significant. It was beneficial for this population to reduce the risk of diabetes and cardiovascular disease by integrating TRE into normal dietary patterns.

## Discussion

To the best of our knowledge, this is the first systematic review of all studies about the efficacy and safety of TRE for patients with prediabetes and diabetes. TRE may be a safe and tolerable dietary intervention for patients with prediabetes and diabetes, and may offer cardiovascular and metabolic benefits.

In the included studies, all studies explored the effect of TRE for weight gain in patients with diabetes and prediabetes, and weight loss was achieved in five studies (11, 12, 15, 16, 19) and not achieved in two studies (17, 18). In total, TRE is an effective intervention for weight loss. A recent study showed TRE was actually greater for weight loss than CR although not significant (20).

For the effect of TRE on insulin sensitivity, four studies were included (11, 12, 16, 18). The measurements of insulin sensitivity were gold standard clamps in two studies (12, 16), OGTT in one study (18), and HOMA-IR in one study (11). Three studies (11, 16, 18) demonstrated that TRE could improve the insulin sensitivity, Sutton’s study (18) found the 6-h TRE could increase insulin sensitivity and improve some aspects of cardiometabolic health for the first time. In Kahleova’s study (16), they found that 10-h TRE increased oral glucose insulin sensitivity (OGIS) and TRE may be a beneficial means for patients with T2DM. In Che’s study (11), they found 10-h TRE improved insulin sensitivity. However, a recent study by Andriessen et al. (12) showed that TRE did not have the effect on insulin sensitivity. The differences in results may be explained by the eating window, the different populations, the measure of insulin sensitivity, and the consumption of the last meal.

All studies explored the effect of TRE on plasma glucose, and a change of plasma glucose was observed in six studies (11, 12, 15–17, 19). Only Sutton’s study (18) was without the change of plasma glucose. The reasons may be an unmatched of fasting duration prior to testing and glucose levels measured only in the morning, and they may have underestimated the glycemic benefits of eTRF, as they mentioned in their study. All studies showed that TRE was a safe, tolerable and feasible intervention for patients with diabetes and prediabetes. In addition, Che et al. (11) demonstrated that TRE could improve the quality of life and reduce the necessary dosage of antidiabetic drugs.

There were some limitations in our study. First, although meta-analysis was planned initially, variation in study designs and different types of TRE, as well as statistical heterogeneity, made the meta-analysis inappropriate, so our study was qualitative rather than quantitative. Second, in our systematic review, seven studies were included, five studies for patients with diabetes and two studies for patients with prediabetes. The studies about TRE for diabetes and prediabetes are limited. Finally, studies with short term may not be informative, and more studies were needed for this field. Some ongoing trials will provide new insight into this field (21–24).

## Conclusion

Time-restricted eating is a safe and feasible intervention, and may offer cardiovascular and metabolic benefits for patients with diabetes and prediabetes. Studies in this field, which should be viewed as important, are limited. Therefore, more high-quality studies are needed.

## Data availability statement

The datasets presented in this study can be found in online repositories. The names of the repository/repositories and accession number(s) can be found in the article/supplementary material.

## Author contributions

SW and JH: study design, research idea, and manuscript writing. YG, GW, and XL: data acquisition, data analysis and interpretation, and statistical analysis. All authors contributed to important work to this study.
